# Editorial: Epigenetic and transcriptional networks underlying ventricular and atrial arrhythmias

**DOI:** 10.3389/fcvm.2022.978891

**Published:** 2022-07-29

**Authors:** Yuanyuan Zhao, Dingsheng Jiang, Yong Xia

**Affiliations:** ^1^Institute of Organ Transplantation, Tongji Hospital, Tongji Medical College, Huazhong University of Science and Technology, Key Laboratory of Organ Transplantation of Ministry of Education, National Health Commission and Chinese Academy of Medical Sciences, Wuhan, China; ^2^Division of Cardiothoracic and Vascular Surgery Tongji Hospital, Tongji Medical College Huazhong University of Science and Technology, Wuhan, China; ^3^Division of Cardiology, Department of Molecular and Cellular Biochemistry, Davis Heart & Lung Research Institute, The Ohio State University, Columbus, OH, United States

**Keywords:** epigenetics, transcriptional regulation, ventricular arrhythmias, atrial arrhythmias, regulatory networks

In the last 30 years, the genetic basis of cardiac electrical phenotypes has been extensively investigated by many research laboratories (1). Recent results have suggested that cardiac electrical diseases, such as ventricular and atrial arrhythmias, are complex traits influenced by a combination of genetic and environmental risk factors that contribute cumulatively to disease predisposition. It is clear that epigenetic modifications such as DNA methylation, histone modifications and non-coding RNA-based mechanisms are the molecular targets for disadvantageous environmental stimuli and may lead to the onset and progression of arrhythmias (2, 3). However, understanding the functional impact of epigenetic and transcriptional networks in arrhythmias is still at its early stage. The main challenge is to elucidate the molecular mechanisms of those epigenetic and transcriptional networks and determine how the changes of these networks give rise to various ventricular and atrial arrhythmias. Such knowledge will further advance the understanding on the pathogenesis of cardiac electrical diseases. This thus becomes the aim of the current special Research Topic. A total of four articles (one Review, one Case Report, and two Researches) have been published in this Research Topic, and the unique contributions of each articles are summarized as follows.

The Review contributed by Li D. et al. provides state-of-the-art view on the epigenetic mechanism of atrial fibrillation (AF) as well as their implications in therapy. AF is the most common arrhythmia and it has a worldwide prevalence of 1.5–2% in the general population (4). In this review, the authors summarized AF associated epigenetic regulation through DNA methylation, histone modifications and chromatin remodeling, and non-coding RNAs. They also discussed epigenetic therapeutic implications in AF. In addition, authors discussed some useful tools such as Chromatin immunoprecipitation (ChIP) and ChIP-seq which were usually used in the study of AF. Finally, authors provided some insights in the budding areas such as using computational model and machine learning to better associate epigenetic changes with AF.

The research by Zhang et al. performed an integrative analysis using PubMed, Medline, China National Knowledge Internet (CNKI), and Wanfang Database to study the genetic characteristics and transcriptional regulation of sodium channel-related genes in Chinese patients with Brugada syndrome (BrS). BrS is an inheritable arrhythmogenic disease which is prone to polymorphic ventricular arrhythmia and sudden cardiac death (5). To date, 23 genes have been found to be related to Brs, and gene SCN5A account for 20–30% (6). In this research, total of 27 suitable studies involving Chinese BrS patients who underwent the SCN5A gene test were included. After analyses, authors found that the distribution of mutations/variations in Brs was region specific in China. Furthermore, post-transcriptional modifications (PTMs) throughout the Nav1.5 protein might be involved in the regulation of the pathogenesis of BrS. In addition to BrS, Danon disease (DD), a rare genetic disorder, was investigated in the Case Report by Li Z. et al.. DD is a glycogen storage lysosomal disorder which usually lead to multisystem syndromes but can also present as cardiac-only symptoms (7). Li Z. et al. reported a novel c.741+2T>C mutation in gene *LAMP2* which caused DD. This mutation led to extra 6-bp preservation of intron 5 at the junction between exons 5 and 6 during transcriptional processing of the LAMP2 mRNA, which creates a stop codon and truncated the LAMP2 protein. Together, these two studies highlighted the roles of genetic characteristics and transcriptional regulation in rare arrhythmogenic diseases.

Finally, another research contributed by Zheng et al. introduced and assessed the diagnostic value of left ventricular mechanical dyssynchrony (LVMD), and the ability to predict major adverse cardiac events (MACE) by using Nitrogen-13 ammonia ECG-gated positron emission tomography (gPET). To date, phase analysis has been thought as an important technique to assess LVMD in diagnosis, therapy, and prognosis evaluation. Authors showed that LVMD from Nitrogen-13 ammonia gated MPI had enhanced diagnostic value for viable myocardium and myocardial scar.

In summary, this Research Topic has provided new insights on the epigenetic and transcriptional regulatory mechanisms of arrhythmogenic diseases such as AF, Brs and DD. Moreover, this Research Topic introduced that LVMD from Nitrogen-13 ammonia gated MPI had diagnostic value for viable myocardium and myocardial scar, which could also be used to detect the function of myocardium in patients with arrhythmia. Together, this collection of Research Topic articles underscores the importance of epigenetic and transcriptional regulation in the development of ventricular and atrial arrhythmia. This new information also suggests new therapeutic targets or strategies to treat these diseases.

## Author contributions

YZ, DJ, and YX wrote and revised the manuscript, have contributed equally to this work, and approved it for publication. All authors contributed to the article and approved the submitted version.
