# Corrigendum: Editorial: Epigenetic and transcriptional networks underlying ventricular and atrial arrhythmias

**DOI:** 10.3389/fcvm.2023.1140389

**Published:** 2023-01-20

**Authors:** Yuanyuan Zhao, Dingsheng Jiang, Yong Xia

**Affiliations:** ^1^Institute of Organ Transplantation, Tongji Hospital, Tongji Medical College, Huazhong University of Science and Technology, Key Laboratory of Organ Transplantation of Ministry of Education, National Health Commission and Chinese Academy of Medical Sciences, Wuhan, China; ^2^Division of Cardiothoracic and Vascular Surgery Tongji Hospital, Tongji Medical College Huazhong University of Science and Technology, Wuhan, China; ^3^Division of Cardiology, Department of Molecular and Cellular Biochemistry, Davis Heart & Lung Research Institute, The Ohio State University, Columbus, OH, United States

**Keywords:** epigenetics, transcriptional regulation, ventricular arrhythmias, atrial arrhythmias, regulatory networks

In the published article, there was an error in affiliations 1, 2, and 3 for first author YZ. The affiliations were incorrectly written as “1, Key Laboratory of Organ Transplantation, Ministry of Education, Chinese Academy of Medical Sciences, Wuhan, China; 2, NHC Key Laboratory of Organ Transplantation, Chinese Academy of Medical Sciences, Wuhan, China; 3, Key Laboratory of Organ Transplantation, Chinese Academy of Medical Sciences, Wuhan, China.”

The correct affiliation is solely:

“1, Institute of Organ Transplantation, Tongji Hospital, Tongji Medical College, Huazhong University of Science and Technology, Key Laboratory of Organ Transplantation of Ministry of Education, National Health Commission and Chinese Academy of Medical Sciences, Wuhan, China.”

The authors apologize for this error and state that this does not change the scientific conclusions of the article in any way. The original article has been updated.

